# Impact of *Opuntia ficus-indica* Juice and Empagliflozin on Glycemic Control in Rats

**DOI:** 10.3390/cimb46110733

**Published:** 2024-11-01

**Authors:** Sondos M. Alqudah, Mohammad Hailat, Zainab Zakaraya, Alaa Azeez Abu Dayah, Mohammad Abu Assab, Samia M. Alarman, Riad M. Awad, Mohammed F. Hamad, Laura Grațiela Vicaș, Wael Abu Dayyih

**Affiliations:** 1 Faculty of Pharmacy and Medical Sciences, University of Petra, Amman 11196, Jordan; phsondosalqudah@gmail.com (S.M.A.); rawad@uop.edu.jo (R.M.A.); 2 Faculty of Pharmacy, Al-Zaytoonah University of Jordan, Amman 11733, Jordan; m.hailat@zuj.edu.jo; 3 Faculty of Pharmacy, Pharmacological and Diagnostic Research Centre (PDRC), Al-Ahliyya Amman University, Amman 19328, Jordan; z.zakaraya@ammanu.edu.jo (Z.Z.); alaa.a.abudayah@gmail.com (A.A.A.D.); 4 Faculty of Pharmacy, Zarqa University, Zarqa 13110, Jordan; mabuassab@zu.edu.jo; 5 Faculty of Pharmacy, Mutah University, Al Karak 61710, Jordan; erman.samia@yahoo.com; 6 Faculty of Medicine, Al-Balqa Applied University, Al-Salt 19117, Jordan; mohammed241168@bau.edu.jo; 7 Department of Pharmacy, Faculty of Medicine and Pharmacy, University of Oradea, 410028 Oradea, Romania; lvicas@uoradea.ro

**Keywords:** *Opuntia ficus*-*indica*, diabetes mellitus, HbA1c, empagliflozin, streptozotocin-induced diabetes, glycemic control

## Abstract

Diabetes mellitus (DM) is a major global health concern characterized by high blood glucose levels. This study investigates the effects of *Opuntia ficus-indica* (cactus) juice and empagliflozin, both alone and in combination, on glycated hemoglobin (HbA1c) levels in healthy and streptozotocin-induced diabetic rats. Eighty Wistar albino male rats were divided into eight groups, with four groups being diabetic. Treatment options included cactus juice, empagliflozin, or both. HbA1c levels were measured at baseline and 100 days later using ELISA. In diabetic and non-diabetic rats treated with cactus juice or empagliflozin, HbA1c levels were significantly reduced, but diabetic rats had significantly lower HbA1c values than non-diabetic rats. The combined treatment provided no additional benefits over individual therapies. These findings indicate that cactus juice and empagliflozin effectively lower HbA1c levels, making their use a promising complementary approach to diabetes management. However, the combined treatment of *Opuntia ficus-indica* juice and empagliflozin did not yield additional reductions in HbA1c levels compared to individual treatments, with no significant synergistic effects observed throughout the study period. More research is needed to better understand the clinical applications and mechanisms in humans.

## 1. Introduction

Diabetes mellitus (DM) is a group of physiological disabilities characterized by hyperglycemia resulting from insulin resistance, insufficient insulin excretion, or elevated glucagon excretion [1,2,3].

Approximately 537 million (10.5%) people (aged 20 to 79) globally manage the condition [4,5]. In Jordan, DM causes about 10% of the annual deaths caused by non-communicable diseases (NCDs). It is estimated that diabetes has a significant impact on health-related economic costs, with approximately 12% (USD 727 billion) of the worldwide medical expenditure being estimated to be caused by diabetes [6].

The management of DM usually pertains to lifestyle changes, including dietary changes, changes in physical activity, and the use of hypoglycemic agents [7,8]. Sodium–glucose co-transporter-2 (SGLT2) antagonists are a novel class of oral anti-diabetic drugs (OADs) [9] that work by lowering glycated hemoglobin levels by raising urinary glucose excretion. Due to their limited ability to reduce blood glucose concentration (BGC), they are typically used in conjunction with metformin and other antihyperglycemic agents, such as insulin, and this treatment comes with the advantages of limited side effects of hypoglycemia and moderate decreases in blood pressure and body weight [10]. In addition, this group of treatments has been shown to lower glycated hemoglobin levels in patients with type 2 diabetes and in those with stage 2 or 3a progressive kidney failure when used as a monotherapy or as an add-on adjunction [11].

The cactus (*Opuntia ficus-India*) is a member of the *Cactaceae* family, also known as the prickly pear. It is a cactus species with a complex taxonomic history. It features ovate-oblong segments, spines, and glochids. It is widely cultivated and typified, with a Linnaean herbarium specimen designated as its neotype. However, its classification has been controversial [12]. This plant is endemic to Mexico and is found across the Mexican and North American regions, Africa, and the Mediterranean basin. It has been used in traditional medicine to treat various diseases and ailments, including diabetes [13]. The extract of *Opuntia ficus-indica* cladodes has been found to have hypoglycemic effects in rabbits, reducing glucose levels by 21.67%. The extract contains polyphenols that regulate glycemia, possibly due to its anti-diabetic activity. The active molecules responsible for the hypoglycemic effect are extracted in water and have polar groupings [14]. *Opuntia ficus-indica* var. *saboten* (OFS) exhibits anti-diabetic effects through enhanced peripheral glucose uptake, activating the AMPK/p38 MAPK pathway [15]. On the other hand, *Opuntia Milpa Alta* extract has also shown significant anti-diabetic effects, particularly through its petroleum ether extract. Further research is needed to understand its mechanism of action and potential long-term effects [16]. *Opuntia ficus-indica* is a long-domesticated cactus crop essential in agricultural economies worldwide, particularly in desert and semi-arid regions. This species’ biogeographic and evolutionary origins have been hidden by ancient and widespread cultivation and naturalization [17]. *Opuntia ficus*-*indica* is used in numerous ways. In modern times, first and foremost, *O. ficus*-*indica* is grown for its large, sweet fruits (often called “tunas”), which are available in local and commercial markets worldwide [18,19]. *Opuntia ficus-indica* is a perennial shrub with a rich bioactive composition, including phenolic acids, flavonoids, carotenoids, amino acids, vitamins, and fibers. It is used in food products, cosmetics, pharmaceuticals, and wastewater treatment. The plant has strong antioxidant activity, antimicrobial resistance, and health benefits. It has potential in nutraceutical development and has commercial value in various industries. Its properties underscore its significance as a food source and a medicinal plant [20].

Additionally, the young cladodes (stem segments) of *O. ficus*-*indica* are harvested as a vegetable crop (often called *nopalitos*). Although this crop is less valuable worldwide than the fruit crop, vegetable products of *O. ficus*-*indica* are available in many local and commercial markets. The medicinal properties of *O. ficus*-*indica* have been documented as early as 1552 [21].

The authors of the present paper reviewed the literature on using *Opuntia ficus-indica* in the treatment of diabetes mellitus. They investigated the effect of a combination of empagliflozin and cactus on glycated hemoglobin HbA1c levels in healthy and streptozotocin-induced diabetic rats, which had not been previously reported.

## 2. Materials and Methods

### 2.1. Preparation of Cactus Juice

*Opuntia ficus-indica* leaves were obtained from a local farm located in Ajloun, in the north of Jordan, and the juice was prepared traditionally by removing the spines, washing the leaves in a pan, juicing the pads using a hummer, tenderizing the pads, and filtering the juice through a wire mesh strainer.

### 2.2. Extraction and UV-HPLC Analysis

Avila-Nava et al. (2014) reported on how to extract flavonoids from cactus leaves using a 70% methanol solution [22]. The mixture was vortexed, sonicated at 37 °C for 10 min, and then kept at room temperature for 50 min with intermittent vortexing. Following centrifugation, a portion of the supernatant was mixed with HCl, rinsed with nitrogen, and incubated in a water bath at 90 °C for 2 h. After acid hydrolysis, the solution was diluted with methanol and centrifuged at 11,000× *g* to perform HPLC-MS analysis.

The ZORBAX Eclipse XDB-C18 reversed-phase column (Agilent Technologies, Santa Clara, CA, USA, 5 µm, 150 mm × 4.6 mm) was used for the analysis. The mobile phase included 0.05% aqueous acetic acid (phase A) and a combination of 0.05% acetic acid in 80% acetonitrile and 20% methanol (phase B), with a flow rate of 0.8 mL per minute. Detection was performed at 280 nm using a DAD detector. Electrospray ionization in negative mode was carried out under the following conditions: nebulizer pressure of 50 psi, dry gas flow rate of 10 L/min, drying temperature of 350 °C, and capillary voltage of 4000 V. The mass spectrum was scanned over the *m*/*z* range of 100–550. The Huang et al. (2009) approach identified and quantified flavonoid peaks, with quercetin acting as an external standard [23]. ChemStation software was used to collect and analyze data (Version B.01.03, Agilent Technologies, Palo Alto, CA, USA). The injection volume was 5.0 μL, and the flow rate was 0.3 mL/min.

### 2.3. Animal Handling

**Scheme 1:**
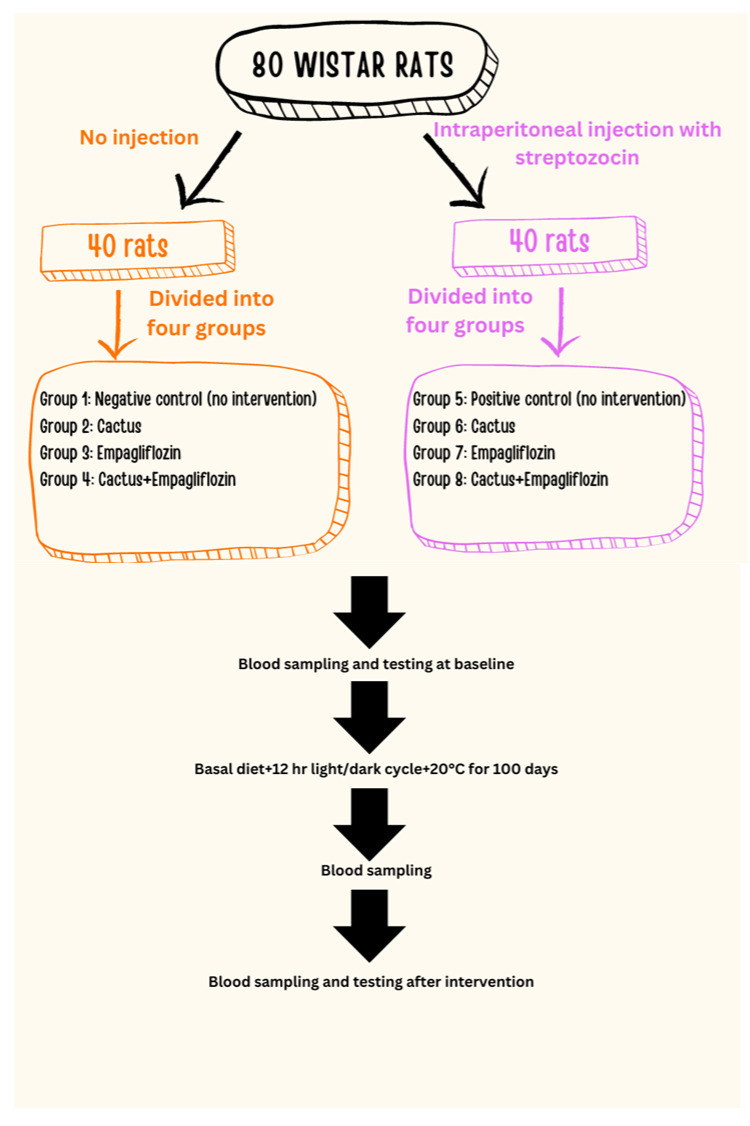
Scheme of experimental procedure and groups.

Eighty healthy Wistar albino male rats were randomly divided into eight groups (*n* = 10 rats/group). The rats, each weighing around 250 g and aged around eight weeks, were delivered to the animal house at Applied Science University. They were placed in a ventilated room with a photoperiod cycle and a temperature of around 20 °C. The eighty rats were divided into eight groups, each with ten rats, as follows (Scheme 1 and Table 1):

### 2.4. Animals and Experimental Procedure

**Figure 1:**
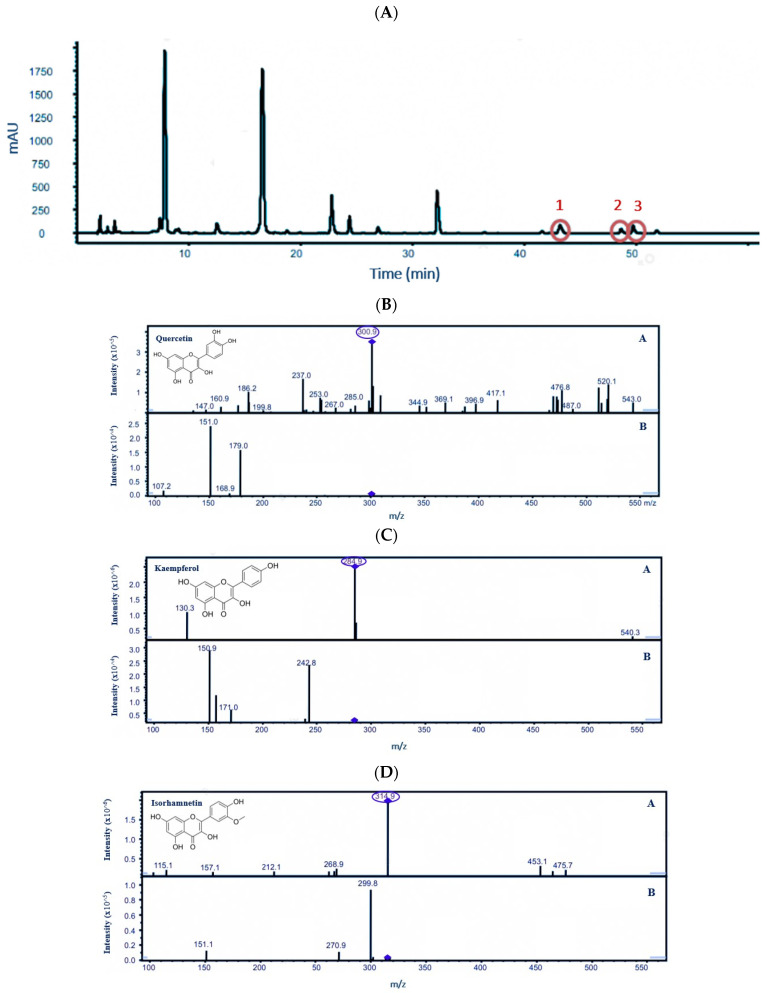
Analysis of the constituents of cactus juice: (**A**) UV-HPLC chromatogram flavonoids (254 nm) of *O. ficus indica*. The identified flavonoids: quercetin (1), kaempferol (2), and isorhamnetin (3). (**B**) MS of ion spectra for quercetin (A: mass spectrum, B: MS/MS fragments). (**C**) MS of ion spectra for kaempferol (A: mass spectrum, B: MS/MS fragments). (**D**) MS of ion spectra for isorhamnetin (A: mass spectrum, B: MS/MS fragments).

Eighty healthy Wistar albino male rats, each aged around eight weeks and weighing around 250 g, were delivered to the animal house at the Applied Science University (Amman, Jordan), were placed in a ventilated temperature- and light-controlled room (temperature around 20 °C, photoperiod cycle), and were randomly divided into eight groups (*n* = 10 rats/group). Four groups were injected with streptozocin to induce diabetes mellitus (75% α-anomer, Millipore-Sigma (Darmstadt, Germany), 3.75 mg/mL sodium acetate buffer) [24], while the other four groups were left intact and were normal. All the rats received a standard diet (including powdered yellow corn, soybean food, DL-methionine, choline chloride, mineral mix, vitamin mix, and corn oil) throughout the experimental period of 100 days and were kept in a room with a 12 h light/dark cycle. For comparison purposes, one group was the negative control group, which was not injected with streptozocin and did not receive any other treatment. Another group served as the positive control group, which was intraperitoneally injected with streptozocin and did not receive any treatment. The remaining three intact groups, as well as the diabetic groups, received the following three different treatments: 5 mL cactus juice orally every 12 h daily; oral gavage with 0.098 mg of empagliflozin (JARDIANCE^®^ tablets (dissolved in water), 0.098 mg/rat [25]) once daily; and 5 mL of cactus juice every 8 h and 0.098 mg of empagliflozin daily by oral gavage. Figure 1 shows the experimental procedure and the experimental groups. Petra and Applied Science Private University institutional guidelines on animal use were followed in the rat handling procedure, which adopted the Federation of European Laboratory Animal Science Association (FELASA) guidelines.

### 2.5. Blood Sample Collection and Analysis

Blood samples were collected from the optical vein of each rat by a capillary tube after 12 h of fasting, at baseline and after 100 days of intervention. Blood samples were kept in a tube collector (EDTA, heparin, and NaF), and the blood samples were incubated for 12 min and analyzed for HbA1c by the Enzyme-Linked Immunosorbent Assay (ELISA) [26].

### 2.6. Statistical Analysis

An unpaired sample *t*-test was used to compare the HbA1C values of diabetic and non-diabetic rats with different treatments. In addition, the mixed effects of time and diabetes, and time and treatment (cactus, empagliflozin, and their combination), were analyzed by a two-way mixed analysis of variance (ANOVA). These were used to compare HbA1c levels, revealing significant differences between treated and control groups over time, affirming the treatments’ efficacy.

## 3. Results and Discussion

This study sought to determine the effects of *Opuntia ficus-indica* juice and empagliflozin on glycemic control in diabetic rats. The study found that both diabetic and non-diabetic rats treated with cactus juice or empagliflozin had significant reductions in HbA1c levels. However, the combination of both did not provide any additional benefits.

### 3.1. Chromatographic Analysis of Cactus Flavonoids

Figure 1 depicts a UV-HPLC chromatogram of flavonoids (254 nm) from *Opuntia ficus-indica*. The identified flavonoids were quercetin (1), kaempferol (2), and isorhamnetin (3). The chromatographic analysis confirms the presence of bioactive compounds in cactus juice that may play a role in glycemic control and are known for their antioxidant properties. 

### 3.2. Glycemic Control in Diabetic and Non-Diabetic Rats

This study utilized a robust experimental design, dividing rats into various treatment groups to isolate the effects of cactus juice and empagliflozin. Table 2 shows that diabetic rats consistently exhibited higher HbA1c levels than their non-diabetic counterparts, underscoring the challenge of managing glycemic levels in diabetic conditions. Table 2 shows the results of the unpaired sample *t*-test for the effect of diabetes on HbA1C values throughout the experimental period. At baseline, no significant differences (*p* < 0.5) were observed between diabetic and non-diabetic rats in HbA1C values across all treatment groups, except for in the case of the combined treatment of cactus and empagliflozin. In this case, non-diabetic rats exhibited significantly lower HbA1C values than the combined treatment diabetic groups (HbA1C values = 5.11 ± 0.37 vs. 5.74 ± 0.35, *p* = 0.001). This indicates that, except for in groups 4 and 8, the allocation of rats into the treatment groups was performed randomly. After that and throughout the experimental period, diabetic rats (regardless of the treatment) exhibited significantly higher (*p* < 0.05) HbA1C values than those of their counter-controls (Table 2). These findings indicate strong anti-diabetic control in *Opuntia ficus-indica* juice and empagliflozin therapies, as both *Opuntia ficus-indica* juice and empagliflozin lowered HbA1c levels in both diabetic and non-diabetic rats, suggesting strong hypoglycemic effects for both treatments. These findings are consistent with the results of other studies [27,28].

Treatment with cactus juice alone showed a significant reduction in HbA1c values as early as ten days into the treatment, suggesting its potential as a natural anti-diabetic agent. This reduction could be attributed to the flavonoids’ action on glucose metabolism pathways. Similarly, empagliflozin, an SGLT2 inhibitor, demonstrated significant hypoglycemic effects, particularly noticeable by the 30th day of treatment. These findings align with the existing literature on the efficacy of SGLT2 inhibitors in lowering HbA1c levels by increasing urinary glucose excretion [29].

### 3.3. Combined Treatment

**Figure 2:**
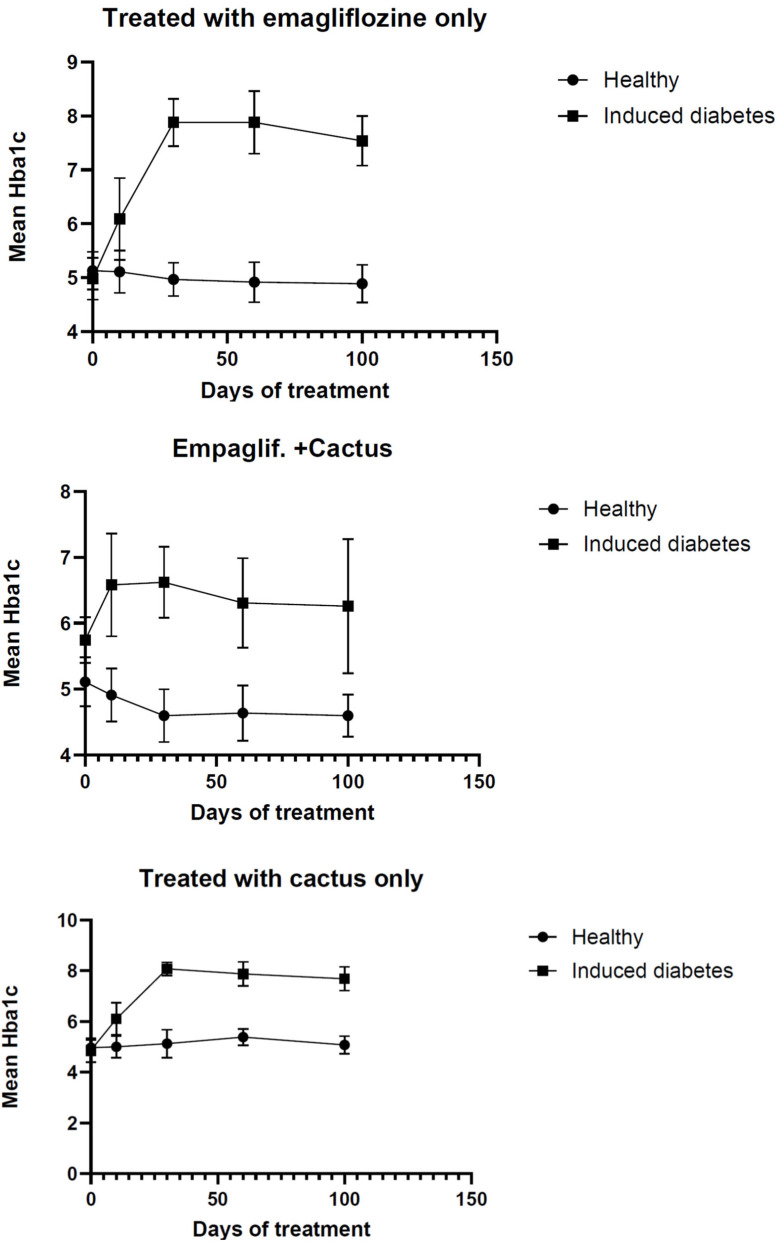
Mean HbA1c levels over time in diabetic and non-diabetic rats with subgroup analysis by treatment type.

**Figure 3:**
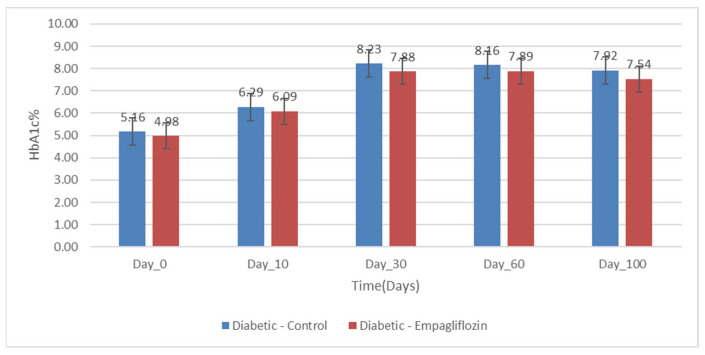
Changes in HbA1c levels over time in rats treated exclusively with empagliflozin.

Figure 2 and Figure 3 show the overall change in HbA1C values between the groups in which diabetic rats exhibited higher HbA1C values than non-diabetic rats. Diabetic rats consistently exhibited higher HbA1c levels compared to non-diabetic controls across all treatment conditions, emphasizing the challenge of maintaining glycemic control in diabetic states. Within the same context, Table 2 shows the mixed effect of time, diabetes, and treatments on the HbA1C values of the study groups. This analysis shows that with time, for rats that did not receive any treatment, there were no significant differences in HbA1C values between diabetic rats and non-diabetic counter-controls. Control rats, rats that received cactus, and those that received empagliflozin exhibited substantial changes in HbA1C ten days into the experimental period, while those that were treated with cactus and empagliflozin showed significant differences from the commencement of the experimental treatment. With time, cactus, empagliflozin, and the combined treatment caused a significant reduction in HbA1C values in both diabetics and their counter-controls.

Interestingly, combining cactus juice and empagliflozin did not result in a synergistic effect. The lack of additional benefits suggests that while both treatments are effective individually, their mechanisms may not complement each other when combined. This finding highlights the importance of understanding drug interactions and the potential for using natural products alongside pharmaceutical agents.

**Figure 4:**
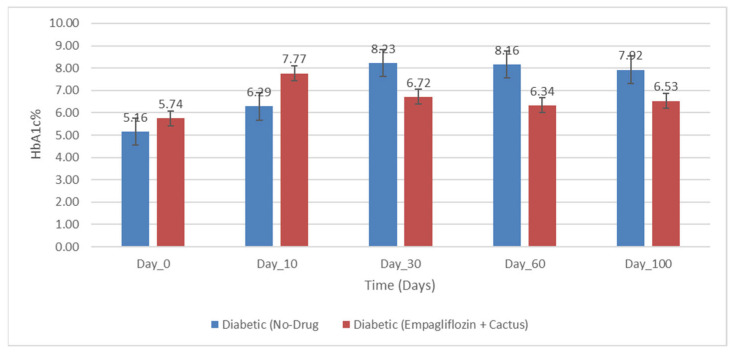
Change in HbA1C values for experimental animals treated with cactus and empagliflozin with time.

In reference to the animal groups that received cactus treatment only, Figure 2 and Figure 3 show that the treatment reduces HbA1C values significantly (*p* < 0.001) in the treated rats as early as ten days into treatment. Nonetheless, on day 30 of treatment, rats exhibited an increase followed by a decrease in HbA1C values. On the other hand, treatment with empagliflozin alone caused a significant (*p* < 0.001) reduction in HbA1C values on the 30th day of administration (Figure 4). The combined therapy of cactus and empagliflozin showed no significant (*p* = 0.111) change in HbA1C values with time in diabetic and non-diabetic rats. Notably, the analysis showed a significant (*p* < 0.001) increase in HbA1C values among diabetic rats who received all treatments compared to non-diabetic rats (Table 2). These results validate the conventional application of *Opuntia ficus-indica* in diabetes management and highlight the effectiveness of empagliflozin as an SGLT2 inhibitor. However, the combined treatment of cactus juice and empagliflozin did not offer additional benefits compared to individual treatments. This indicates no synergistic effect between the two therapies.

## 4. Conclusions

This study highlights the effectiveness of *Opuntia ficus-indica* juice and empagliflozin as complementary diabetes treatments. It suggests incorporating *Opuntia ficus-indica* into standard treatment regimens and flavonoids from cactus juice into standard treatments to control HbA1c levels. However, further research is needed to understand these treatments’ underlying mechanisms and clinical applications in human diabetes management. Their combination’s lack of additive benefits underscores the need for further investigation.

## Figures and Tables

**Table 1:** Rat grouping and the assigned treatments.

Group #1	No drug	In this control group of healthy rats, no medication was given, and the food given was basic and regular daily food. This group remained in this study for about 12 weeks under the same environmental conditions.
Group #2	Cactus	Healthy rats were given 5 mL of cactus juice every 8 h daily for 12 weeks, and no medication was given.
Group #3	Empagliflozin	Healthy rats were given 0.107 mg of empagliflozin once daily for 12 weeks using suitable oral gavage and given a normal basal diet with water.
Group #4	Cactus+ empagliflozin	Healthy rats were given 5 mL of cactus juice every 8 h and 0.107 mg of empagliflozin using suitable oral gavage.
Group #5	No drug	Diabetic rats were given a normal basal diet with water.
Group #6	Cactus	Diabetic rats were given 5 mL of cactus juice every 8 h daily for 12 weeks, and no medication was given.
Group #7	Empagliflozin	Diabetic rats were given 0.107 mg of empagliflozin once daily for 12 weeks using suitable oral gavage and given a normal basal diet with water.
Group #8	Cactus+ empagliflozin	Diabetic rats were given 5 mL of cactus juice every 8 h and 0.107 mg of empagliflozin using suitable oral gavage.

**Table 2:** Bivariate analysis of HbA1c levels in diabetic and non-diabetic rats. The analysis was conducted at baseline and after 10, 30, 60, and 100 days of treatment with cactus juice, empagliflozin, and their combinations. Unpaired samples *t*-test (*n* = 10).

Treatment Type	Group#	Animal Disease State	Baseline	At 10 Days	At 30 Days	At 60 Days	At 100 Days
			Mean (SD)	Mean (SD)	Mean (SD)	Mean (SD)	Mean (SD)
No Treatment	1	Healthy	5.20 (0.35)	5.24 (0.41)	5.21 (0.34)	5.18 (0.35)	5.09 (0.31)
	5	Induced Diabetes	5.16 (0.42)	6.29 (0.65)	8.23 (0.35)	8.16 (0.56)	7.92 (0.50)
		*p*-Value	*p* = 0.820	*p* < 0.001	*p* < 0.001	*p* < 0.001	*p* < 0.001
Cactus Only	2	Healthy	4.97 (0.31)	5.00 (0.43)	5.13 (0.55)	5.39 (0.33)	5.08 (0.35)
	6	Induced Diabetes	4.87 (0.47)	6.10 (0.64)	8.08 (0.26)	7.88 (0.48)	7.69 (0.47)
		*p*-Value	*p* = 0.568	*p* < 0.001	*p* < 0.001	*p* < 0.001	*p* < 0.001
Empagliflozin Only	3	Healthy	5.13 (0.35)	5.11 (0.39)	4.97 (0.31)	4.92 (0.37)	4.89 (0.35)
	7	Induced Diabetes	4.98 (0.39)	6.09 (0.76)	7.88 (0.44)	7.88 (0.58)	7.54 (0.46)
		*p*-Value	*p* = 0.367	*p* = 0.002	*p* < 0.001	*p* < 0.001	*p* < 0.001
Combined Empagliflozin and Cactus	4	Healthy	5.11 (0.37)	4.91 (0.40)	4.60 (0.40)	4.64 (0.42)	4.60 (0.32)
	8	Induced Diabetes	5.74 (0.35)	6.58 (0.78)	6.62 (0.54)	6.31 (0.68)	6.26 (1.02)
		*p*-Value	0.001	<0.001	<0.001	0.001	0.001

## Data Availability

Data can be obtained from the authors.

## References

[1] Rahman M.R., Islam T., Nicoletti F., Petralia M.C., Ciurleo R., Fisicaro F., Pennisi M., Bramanti A., Demirtas T.Y., Gov E. (2021). Identification of Common Pathogenetic Processes between Schizophrenia and Diabetes Mellitus by Systems Biology Analysis. Genes.

[2] Sweidan N.I., Abu Khalaf R.A., Shatat A.M., Hammad W.A. (2022). Therapeutic Potential of Silybum Marianum and Pergularia Tomentosa Extracts from Jordanian Origin in Diabetes Mellitus. Curr. Bioact. Compd..

[3] Dayyih W.A., Hailat M.M., Zakareia Z., Abu Dayyih W.A., Manaysa M.H., Hailat M.M., Zakareia Z., El Hajji F., Dayyih W.A., Manaysa M.H. (2021). Influence of Castor Oil on Glycated Hemoglobin (Hba1c) on Induced Type 2 Diabetes Mellitus in Rats. Jordan J. Pharm. Sci..

[4] Diabetes. https://www.who.int/health-topics/diabetes#tab=tab_1.

[5] Tamimi L.N., Zakaraya Z., Hailat M., Abu Dayyih W., Daoud E., Abed A., Saadh M.J., Majeed B., Abumansour H., Aburumman A. (2023). Anti-Diabetic Effect of Cotreatment with Resveratrol and Pioglitazone in Diabetic Rats. Eur. Rev. Med. Pharmacol. Sci..

[6] Shirai Y., Imai T., Sezaki A., Miyamoto K., Kawase F., Abe C., Sanada M., Inden A., Kato T., Suzuki N. (2021). Trends in Age-Standardised Prevalence of Type 2 Diabetes Mellitus According to Country from 1990 to 2017 and Their Association with Socioeconomic, Lifestyle and Health Indicators: An Ecological Study. J. Glob. Health.

[7] Marín-Peñalver J.J., Martín-Timón I., Sevillano-Collantes C., del Cañizo-Gómez F.J. (2016). Update on the Treatment of Type 2 Diabetes Mellitus. World J. Diabetes.

[8] Mosleh R.S., Abd Aziz N., Ali S.M., Manan M.M., Zyoud S., Shah I.M., Jarrar Y.B. (2017). Predictors of Good Glycemic Control among Type II Diabetes Patients in Palestine. Asian J. Pharm. Clin. Res..

[9] Kalra S. (2014). Sodium Glucose Co-Transporter-2 (SGLT2) Inhibitors: A Review of Their Basic and Clinical Pharmacology. Diabetes Ther..

[10] Reddy R.P.M., Inzucchi S.E. (2016). SGLT2 Inhibitors in the Management of Type 2 Diabetes. Endocrine.

[11] Vivian E.M. (2014). Sodium-Glucose Co-Transporter 2 (SGLT2) Inhibitors: A Growing Class of Antidiabetic Agents. Drugs Context.

[12] Ginestra G., Parker M.L., Bennett R.N., Robertson J., Mandalari G., Narbad A., Lo Curto R.B., Bisignano G., Faulds C.B., Waldron K.W. (2009). Anatomical, Chemical, and Biochemical Characterization of Cladodes from Prickly Pear [*Opuntia ficus-indica* (L.) Mill.]. J. Agric. Food Chem..

[13] Kaur M., Kaur A., Sharma R. (2012). Pharmacological Actions of *Opuntia ficus indica*: A Review. J. Appl. Pharm. Sci..

[14] Halmi S., Benlakssira B., Bechtarzi K., Djerrou Z., Djeaalab H., Riachi F., Pacha Y.H. (2012). Antihyperglycemic Activity of Prickly Pear (*Opuntia ficus-indica*) Aqueous Extract. Int. J. Med. Arom. Plants.

[15] Leem K.H., Kim M.G., Hahm Y.T., Kim H.K. (2016). Hypoglycemic Effect of *Opuntia ficus-indica* Var. Saboten Is Due to Enhanced Peripheral Glucose Uptake through Activation of AMPK/P38 MAPK Pathway. Nutrients.

[16] Zhang W., Sheng C., Zheng C., Yao J. (2010). Chemical Composition and Antidiabetic Activity of Opuntia Milpa Alta Extracts. Chem. Biodivers..

[17] Choudhary M., Kumar R., Neogi S. (2020). Activated Biochar Derived from *Opuntia ficus-indica* for the Efficient Adsorption of Malachite Green Dye, Cu^2+^ and Ni^2+^ from Water. J. Hazard. Mater..

[18] Basile Professor of Agriculture F. (2001). Economic Aspects of Italian Cactus Pear Production and Market. J. Prof. Assoc. Cactus Dev..

[19] Inglese P., Basile F., Schirra M. (2012). Cactus Pear Fruit Production. Cacti: Biology and Uses.

[20] Aruwa C.E., Amoo S.O., Kudanga T. (2018). Opuntia (Cactaceae) Plant Compounds, Biological Activities and Prospects—A Comprehensive Review. Food Res. Int..

[21] Mounir B., Asmaa M., Abdeljalil Z., Abdellah A. (2020). Physico-Chemical Changes in Cladodes of *Opuntia ficus-indica* as a Function of the Growth Stage and Harvesting Areas. J. Plant Physiol..

[22] Avila-Nava A., Calderón-Oliver M., Medina-Campos O.N., Zou T., Gu L., Torres N., Tovar A.R., Pedraza-Chaverri J. (2014). Extract of Cactus (*Opuntia ficus indica*) Cladodes Scavenges Reactive Oxygen Species in Vitro and Enhances Plasma Antioxidant Capacity in Humans. J. Funct. Foods.

[23] Huang Z., Wang B., Eaves D.H., Shikany J.M., Pace R.D. (2009). Total Phenolics and Antioxidant Capacity of Indigenous Vegetables in the Southeast United States: Alabama Collaboration for Cardiovascular Equality Project. Int. J. Food Sci. Nutr..

[24] Ghasemi A., Jeddi S. (2023). Streptozotocin as a Tool for Induction of Rat Models of Diabetes: A Practical Guide. EXCLI J..

[25] Akbarzadeh A., Norouzian D., Mehrabi M.R., Jamshidi S.H., Farhangi A., Verdi A.A., Mofidian S.M.A., Rad B.L. (2007). Induction of Diabetes by Streptozotocin in Rats. Indian J. Clin. Biochem..

[26] Parasuraman S., Raveendran R., Kesavan R. (2010). Blood Sample Collection in Small Laboratory Animals. J. Pharmacol. Pharmacother..

[27] Elshehy H., Salah S., Abdel-Mawla E., Agamy N. (2020). Protective Effect of *Opuntia ficus-indica* Against Diabetes in Alloxan-Induced Diabetic Rats. Can. J. Clin. Nutr..

[28] Abd El-Razek F., El-Metwally E., Shehab G.M.G., Hassan A., Gomaa A. (2012). Effects of Cactus Pear (*Opuntia ficus indica*) Juice on Oxidative Stress in Diabetic Cataract Rats. Saudi J. Health Sci..

[29] Scheen A.J. (2015). SGLT2 Inhibition: Efficacy and Safety in Type 2 Diabetes Treatment. Expert Opin. Drug Saf..

